# The burden of breast cancer in Poland: current status and future directions

**DOI:** 10.3389/fonc.2025.1599741

**Published:** 2025-06-26

**Authors:** Wojciech Sierocki, Ligia Kornowska, Alicja Dudek, Małgorzata Szpakowicz, Claudia Dompe, Magdalena Roszak

**Affiliations:** ^1^ Department of Continuing Education, University of Oxford, Oxford, United Kingdom; ^2^ Department of Pathophysiology, Poznan University of Medical Sciences, Poznan, Poland; ^3^ Nuffield Department of Primary Care Health Sciences, Oxford, United Kingdom; ^4^ Department of Data Analytics, Fundacja Podaruj Dane, Warsaw, Poland; ^5^ ​​Second Department of General Surgery, Jagiellonian University Medical College, Kraków, Poland; ^6^ Department of Endocrinology Centrum Medycznego Kształcenia Podyplomowego (CMKP), Bielanski Hospital in Warsaw, Warsaw, Poland; ^7^ Department of Cancer Epidemiology and Primary Prevention, Maria Sklodowska-Curie National Research Institute of Oncology, Warsaw, Poland; ^8^ Department of Immunology, Poznan University of Medical Sciences, Poznan, Poland; ^9^ Doctoral School, Poznan University of Medical Sciences, Poznan, Poland

**Keywords:** breast cancer, national health fund, epidemiology, prevention, treatment, Poland

## Abstract

Breast cancer poses a significant public health challenge in Poland, with incidence rates exceeding the EU average and a concerning disparity between incidence and mortality. This review analyzes the key steps of breast cancer diagnosis and treatment in Poland, examining factors influencing therapeutic outcomes. We explore the complex interplay of epidemiological trends, prevention strategies, the impact of the National Oncology Strategy (NSO) and the National Cancer Network (KSO), access to care challenges, treatment protocols, and drug programs. While Poland has made strides in implementing comprehensive care pathways, including the DiLO card system and dedicated drug programs, significant gaps remain. Low screening participation, delays in diagnosis, limited access to innovative therapies, and reimbursement restrictions hinder optimal patient outcomes. This review highlights the urgent need for targeted interventions to address these challenges, improve early detection, enhance access to care, and ultimately reduce the burden of breast cancer for Polish patients.

## 1 Introduction

Malignant tumors remain a significant public health concern worldwide, with the International Agency for Research on Cancer (IARC) projecting a dramatic increase in new cases, estimating over 35 million annually by 2050, a nearly 80% rise from the 20 million cases in 2022 (1). This alarming rise is driven by factors such as an aging global population, increased exposure to modifiable lifestyle risks (e.g., tobacco use, physical inactivity, excessive alcohol consumption, overweight/obesity), and growing exposure to environmental hazards, particularly air pollution. In 2022, breast cancer was the most commonly diagnosed cancer in women worldwide, accounting for 2.3 million new cases and 670,000 deaths (2). This translates to the unfortunate reality of it being the most frequent cancer in women across 158 of 185 countries, including Poland (3). According to the latest data from the National Cancer Registry (Krajowy Rejestr Nowotworów - KRN), for 2022, there were 21,554 cases and 6,611 deaths attributable to breast cancer among women, representing 23.6% of all registered female cancer cases and 14.9% or 15% of female cancer deaths (4, 5).

Breast cancer originates from the epithelium of the ducts or lobules of the mammary gland and is influenced by several risk factors, including female gender, age (risk increases with age), genetic factors (family history, particularly in first-degree relatives, and gene mutations like BRCA1 and BRCA2), hormonal factors (early menarche, late menopause, nulliparity or late first pregnancy, oral contraceptive use, and hormone replacement therapy), obesity, physical inactivity, poor diet, and excessive alcohol consumption (6, 7). However, a striking reality is that approximately half of all cases occur in women without any identifiable risk factors beyond being female and over 40 (2). This highlights the complex interplay of genetic, environmental, and lifestyle factors and underscores the need for ongoing research into the disease’s underlying mechanisms.

Significant efforts are needed to improve both early detection and treatment outcomes for breast cancer. Despite this need, data from the National Health Fund (Narodowy Fundusz Zdrowia – NFZ) reveal that only 37% of eligible Polish women have participated in the national mammography screening program (8). This falls far short of the recommended 70% participation rate necessary to achieve a meaningful population-level reduction in breast cancer mortality. This low uptake is particularly concerning given Poland’s relatively high breast cancer mortality rate (41.3 deaths/100,000) compared to its incidence rate in the general population (117.8 cases/100,000) within the EU (9). This disparity between incidence and mortality highlights weaknesses in early detection, such as low screening rates, delays in diagnosis, or limited access to diagnostic tools. Furthermore, it could also point to challenges in treatment effectiveness, including access to optimal therapies, adherence to treatment protocols, or the prevalence of more aggressive tumor subtypes. Understanding the specific contributors to this disparity is crucial for developing targeted interventions.

This review aims to analyze the key steps of breast cancer diagnosis and treatment in Poland, exploring the factors influencing therapeutic outcomes. By examining these factors, including screening practices, diagnostic pathways, treatment protocols, and patient-related factors, we hope to identify opportunities for improvement in the care and outcomes for Polish women affected by this disease. The presented epidemiological data underscores the urgency and importance of this work.

## 2 Breast cancer burden in Poland

Tumors represent substantial and escalating health, social, and economic challenges in Poland, a problem projected to intensify due to significant demographic shifts (10). The Central Statistical Office (Główny Urząd Statystyczny -GUS) predicts a dramatic increase in the number of people over 60, potentially reaching 13.7 million by 2050—a staggering 40% of the population (11, 12). This demographic shift is deeply concerning, as cancer incidence strongly correlates with age, foreshadowing a significant surge in cases and a crushing burden on the healthcare system (10). In 2022 alone, cancer claimed 96,062 lives in Poland, making it the second leading cause of death, and over 180,000 new cancer diagnoses were made, an incidence rate for the general population of 486.1 cases per 100,000 people (4). Among women, breast cancer is the most prevalent malignancy, representing nearly a quarter of all cancers diagnosed (4). Regardless of the elevated number of new cases diagnosed annually, and despite medical advances and increased awareness, breast cancer remains a formidable foe.

The complex nature of cancer care translates into enormous medical and social costs across Europe, and Poland is no exception. Poland’s spending on cancer treatment has skyrocketed, surpassing PLN 10 billion annually since 2019 and reaching PLN 15.5 billion (€90 per person) in 2022 (13). While this substantial investment demonstrates commitment, it still falls significantly short of the 2018 European average of €195 per person. Although hospitalization services comprise the bulk of oncology expenditure, the fastest-growing cost component is spending on increasingly vital drugs within drug programs (13). Within this broader context, breast cancer poses a substantial economic burden in Poland, encompassing both direct and indirect costs, with significant implications for the healthcare system and broader economy. In 2019, the Annual Present Value of Future Lost Productivity (PVFLP) due to breast cancer mortality in Poland was estimated at €85 million, the highest among nine Central and Eastern European countries (14). More comprehensive analyses, including absenteeism and disability, suggest even higher productivity losses (15, 16). Total expenditures for breast cancer in Poland rose from €305,371,000 in 2017 to €344,649,000 in 2019, with indirect social costs from absenteeism increasing to over 30% of total expenditures (17). This increase reflects a rise in both sick leave days and medical certificates. It’s crucial to note that while this study focused on absenteeism, a broader analysis indicates that absenteeism accounts for only about 18% of total social costs, implying the true indirect costs could be significantly higher, potentially exceeding direct costs when factors like presenteeism, caregiver costs, premature mortality, and disability are included. Annually, breast cancer treatment costs the state budget approximately PLN 500 million.

With rising cancer mortality, an aging population, and escalating treatment costs, optimizing oncology treatment standards and carefully monitoring outcomes in Poland is more critical than ever. Inefficient diagnostic and treatment pathways not only strain the healthcare budget but, far more importantly, jeopardize patient health and lives. Improving the efficiency and effectiveness of cancer care in Poland is, therefore, not just a fiscal imperative but a fundamental matter of life and death. Prevention programs are crucial in the fight against cancer, with their effectiveness largely dependent on screening participation rates (18). Early detection of breast cancer significantly improves treatment options and reduces mortality (19). Poland’s Breast Cancer Prevention Program offers regular free mammograms to women, aged 45-74, in three categories: those who haven’t had a mammogram in two years; those who have completed five years of surgical breast cancer treatment and are on hormone replacement therapy; and those who have completed breast cancer treatment and five years of post-treatment monitoring (20). Despite numerous educational and promotional efforts, enrolment in these programs remains insufficient in Poland (21). National mammography rates for eligible women in Poland (around 50%) are considerably below the European Union average (around 70%) (22). The situation highlights the urgent need to address the factors hindering participation in breast cancer screening programs in Poland.

## 3 The national oncology strategy

Observation of highly developed countries that have introduced the so-called “Cancer Plan” shows that integrating activities in prevention, scientific development, education, system changes, and technology transfer leads to significant improvements in epidemiological indicators (23). In Poland, cancer is one of the biggest health challenges, as the number of cancer cases is steadily increasing. Therefore, it is necessary to carry out integrated, systemic activities that target malignant tumors.

Recognizing the need for a coordinated and comprehensive response, the country has embarked on a transformative journey in oncology care through the implementation of the National Oncology Strategy (Narodowa Strategia Onkologiczna - NSO) for 2020-2030 (24). This ambitious, multi-year program aims to significantly improve cancer outcomes by focusing on five key strategic areas: strategic investment in healthcare personnel, enhanced education and training, a patient-centric approach, advancements in science and innovation, and a robust and adaptable oncology care system. The overarching goals of the NSO are to increase the 5-year survival rate for cancer patients following the completion of their primary treatment, to improve the early detection of cancer, and to enhance the overall quality of life for patients both during their cancer journey and in the crucial period following treatment (25). The NSO’s objectives encompass a broad range of initiatives, including reducing the overall incidence of cancer through targeted health education campaigns, proactive health promotion activities, and comprehensive prevention strategies, including fostering pro-health awareness and promoting healthy lifestyles. A core focus is improving the entire spectrum of cancer care, from prevention and early detection to accurate diagnosis and effective treatment. The NSO seeks to develop a patient-centered healthcare system in oncology, placing the patient and their needs at the forefront of all activities, with particular emphasis on improving the quality of life for patients and their families. Ensuring equal access to high-quality healthcare services in oncology, delivered in accordance with the most current medical knowledge, is another key objective. Finally, the NSO aims to implement organizational changes to provide patients equal access to coordinated and comprehensive healthcare across oncology care (26).

The NSO made significant strides, focusing on prevention, advanced treatments, and stronger healthcare infrastructure. With a 420.5 million PLN budget (part of a 5.1 billion PLN total allocation), the NSO completed 63 of 92 planned tasks, with the annual budget increasing to 500 million PLN in 2024 (27). Key achievements included the implementation of digital mammography across screening programs, an important step forward in early breast cancer detection. Nearly 345 million PLN also funded vital equipment like mammographs (28). A specialized program designed to care for families with a high hereditary risk of developing selected malignancies, including breast cancer, was introduced (29, 30). The range of innovative therapies covered by reimbursement was expanded, including many oncology-related molecules. Beyond diagnostics and treatment, the NSO also prioritized medical education and training, including updates to curricula and specialization programs, aiming to ensure a well-prepared oncology workforce. Additionally, public health campaigns, such as the “I Plan a Long Life” (“Planuję długie życie”) initiative, promote awareness and encourage healthy lifestyle choices (31).

## 4 National cancer network (KSO)

A cornerstone of the NSO is the establishment of the National Cancer Network (Krajowa Sieć Onkologiczna – KSO) (32). The KSO’s new oncology care model aims to provide standardized, comprehensive care to all Polish cancer patients, regardless of location. This initiative supports the NSO by optimizing care organization, improving quality, safety, and patient satisfaction, while also controlling costs and improving cancer outcomes.

The KSO’s structural foundation will be comprised of Specialized Oncology Treatment Centers (Specjalistyczne Ośrodki Leczenia Onkologicznego - SOLO), operating at three distinct levels: SOLO III (highly specialized/centers of excellence), SOLO II (complex services), and SOLO I (basic services). Additionally, all SOLO centers will adhere to standardized protocols and interdisciplinary collaboration, and only KSO-qualified entities will be authorized to provide publicly funded oncology care (33). Participating KSO entities will assign a patient coordinator to support patients throughout their oncology journey. While the coordinators will prepare patients for consultations, schedule appointments and tests, and facilitate communication between patients and physicians, systematic evaluations of patient satisfaction will be performed. This feedback mechanism will allow for continuous monitoring of the system’s effectiveness and enable necessary adjustments and improvements to be implemented (33). Within the framework of the KSO, the National Monitoring Center (Krajowy Ośrodek Monitorujący - KOM) is operated by the Maria Sklodowska-Curie National Institute of Oncology - National Research Institute (NIO-PIB) in Warsaw, and it develops and updates national oncology diagnostic and therapeutic guidelines (34). The NIO-PIB is actively cooperating with The National Comprehensive Cancer Network, a globally recognized organization developing evidence-based cancer care guidelines (35). This collaboration ensures that the guidelines developed and implemented in Poland are adapted to the specific conditions and resources of the Polish healthcare system, maximizing their relevance and effectiveness.

## 5 Access to care

An important trend in modern healthcare systems is Person-Centered Care, which prioritizes patients by providing access to information, respecting their dignity, and enabling participation in treatment decisions. This approach positions the patient as a partner in their care and rehabilitation. Studies have demonstrated that Person-Centered Care leads to improved health outcomes, more efficient resource utilization, lower costs, and increased patient satisfaction, ultimately resulting in better adherence to medical recommendations and treatment plans, faster recovery, and reduced risk of complications (36, 37).

Effective access to diagnostic and treatment services is a fundamental prerequisite for realizing person-centered cancer care. Without timely access, patients cannot fully participate in their care or benefit from the latest advancements in treatment. Poland has implemented several strategies to improve access to oncology care, including the oncology fast track facilitated by the Diagnostic and Oncological Treatment Card (karta Diagnostyki i Leczenia Onkologicznego - DiLO) card (38). While the number of issued DiLO cards has increased annually, the percentage of cards resulting in timely diagnoses has unfortunately decreased. In the first half of 2023, only 65% of initial diagnosis cards and 71% of in-depth diagnosis cards led to timely diagnoses, compared to 76% for both levels in 2018 (39). Additionally, differences between provinces are still evident, varying between 45% to 90%.

Despite increasing registered medicinal products and reimbursed oncology therapies, access to modern anticancer drugs remains limited in Poland. The rapid growth in the number of drug technologies registered by the European Medicines Agency and recommended by the European Society of Clinical Oncology (ESMO) presents a challenge to the Polish reimbursement system (39). For example, in the case of breast cancer, the waiting time for drug reimbursement after registration in Poland can be as long as 1,000 days (40). While access to innovative therapies and diagnostic solutions in Poland improved by 5 points in 2023, reaching 58 out of 100 on the key performance indicators (KPI) scale (38), the overall access gap in Poland is rated at 64% (41). This gap is largely attributed to limited reimbursement and restrictive qualification criteria. Specifically, Poland’s reimbursement rate of 46% falls below the EU average of 50%, and notably, while a staggering 76% of reimbursed therapies in Poland are subject to restrictions, ranking among the highest in the compared countries (39). The W.A.I.T. (Waiting to Access Innovative Therapies) indicator, a prominent European tool for measuring innovative drug availability and patient wait times, reveals that Poland ranks 22nd out of 37 analyzed countries in overall cancer therapy availability. The average time for new breast cancer drugs to be reimbursed in Poland is almost 3.1 years (42). These combined indicators demonstrate that many Polish patients still lack access to optimal cancer treatments.

## 6 Treatment

Breast cancer treatment in Poland is structured around a comprehensive, interdisciplinary, and patient-centered approach. It is aligned with the ESMO guidelines, which state that treatment depends on tumor stage, subtype, hormone receptor presence, and HER2 protein presence (43). The Polish system emphasizes rapid diagnosis, individualized treatment plans, and coordinated care throughout the patient journey. Breast Cancer Units (BCUs) aim to improve the treatment results and provide comprehensive diagnostics, specialized expertise (clinical oncology, cancer surgery, radiation therapy, pathomorphology), psycho-oncological support, and rehabilitation. A key element of the Polish system is the “fast track” for oncology, facilitated by the DiLO card. If a primary care physician suspects breast cancer, they issue a DiLO card, referring the patient to a specialist (44, 45). Additionally, a doctor providing outpatient specialty services, in the case of suspected malignant neoplasm, can also issue a DiLO card. This initiates a process of preliminary diagnostics, typically including breast ultrasound and/or mammography, which should be completed within 28 days (46). If cancer is confirmed, the patient proceeds to in-depth diagnostics to determine cancer type, stage, and metastases. These diagnostics may include biopsy, MRI, CT scans, scintigraphy, molecular and genetic tests, and PET scans, and should be completed within 21 days (46).

Following diagnostics, the next step is a medical consultation, during which a decision is made on the treatment method and its schedule. A multidisciplinary team of specialists takes part in the consultation, including an oncologist, a radiotherapist, a surgeon, and a radiologist (47). In addition, a psychologist, a nurse, or another medical professional and the patient may also participate. During the consultation, a coordinator is assigned to accompany the patient until the end of the treatment, providing informational, administrative, and organizational support, including assistance in communication between the patient and the therapeutic team. The time for the meeting of the council and the start of the agreed treatment should not exceed 2 weeks from the date of the patient’s admission to the hospital (44). Treatment in Poland, following ESMO guidelines, often involves a combination of methods, such as surgery (sparing surgery or mastectomy), radiation therapy, chemotherapy, hormone therapy, and targeted therapies, and a specific combination is tailored to each patient (43).

The treatment path is personalized, considering the tumor’s biological characteristics, previous treatment results, disease severity, the patient’s overall health, and, importantly, the patient’s preferences. Treatment may involve multiple lines, with the physician adjusting the plan based on the patient’s response (48). After treatment, the coordinator transfers the patient to a specialist. If tests show no deterioration, the patient returns to their primary care physician for long-term care, guided by an ongoing monitoring program (45). Additionally, the hospital must supply comprehensive treatment to the patient, even in cooperation with other providers. The Polish system, utilizing the DiLO card, the medical consultation team, patient coordinators, and BCUs, aims to provide a coordinated, efficient, and patient-focused approach to breast cancer treatment, aiming for optimal outcomes and improved quality of life in accordance with ESMO guidelines.

## 7 Drug programs

Poland’s commitment to providing access to innovative, reimbursable therapies for breast cancer is manifested in the dedicated drug program B.9.FM “Treatment of patients with breast cancer,” the largest and most cost-intensive oncology program in the country. The program’s structure ensures a personalized approach to treatment, with reimbursed therapeutic options tailored to the specific stage and subtype of breast cancer. For instance, patients diagnosed with HER2-positive breast cancer have access to Lapatinib, Pertuzumab, Trastuzumab, Trastuzumab emtansine, Tucatinib, and Trastuzumab deruxtecan. Conversely, those with HER2-negative breast cancer are offered Palbociclib, Ribociclib, Abemaciclib, Alpelisib, Talazoparib, and Olaparib (49). Similarly, patients facing the challenge of triple-negative breast cancer can access Pembrolizumab, Sacituzumab Govitecan, Talazoparib, and Olaparib through the program.

The financial commitment to breast cancer treatment in Poland further underscores the program’s importance. In 2022, the NFZ allocated approximately PLN 7.2 billion for all drug programs nationwide, and a significant portion of this allocation was dedicated to breast cancer treatment under B.9.FM (50). This considerable allocation demonstrates the program’s reach and impact. Looking ahead, preliminary figures reveal continued strong support for B.9.FM, building upon substantial expenditures in recent years, showcasing a consistent and growing financial dedication to this critical area of healthcare. Despite this considerable investment and the breadth of therapies available under B.9.FM, limitations persist in accessing the full range of treatments recommended by international guidelines. A 2023 assessment revealed that out of 18 therapies recommended by the ESMO for breast cancer, only five were fully funded in Poland in accordance with the standards (13). An additional six therapies were available, albeit with limitations, while seven drug technologies endorsed by clinical management guidelines remained entirely unreimbursed. Therefore, while drug program B.9.FM represents a significant stride in providing breast cancer treatment in Poland, offering a variety of innovative therapies tailored to different cancer subtypes and demonstrated by substantial financial commitment, the identified gaps between available treatments and international recommendations highlight a crucial area for improvement. Addressing these disparities is of utmost importance to continue enhancing outcomes for breast cancer patients in Poland.

## 8 Conclusions

While Poland has made notable strides in breast cancer care through initiatives like the NSO, KSO, and dedicated drug programs, significant challenges remain. The persistent disparity between breast cancer incidence and mortality underscores the need for intensified efforts to improve early detection, streamline diagnostic pathways, expand access to innovative therapies, and strengthen patient-centered care. Addressing these challenges through targeted interventions, continuous evaluation, and a commitment to research will be crucial to reducing the burden of breast cancer and improving outcomes for Polish patients. Access to good-quality health data would be crucial in designing and monitoring future interventions. In a promising development, Poland recently allocated over €100 million to establish 19 Regional Digital Medicine Centres (RDMCs) nationwide. This substantial investment, spearheaded by the Polish Medical Research Agency (ABM), aims to bolster digital infrastructure for clinical research, data analysis, and broader healthcare innovation (51). Embracing principles of data altruism and data donation, the routine capture of health data through an opt-in mechanism could provide an invaluable source of information for future analyses and advancements in care, particularly within oncology.
